# Evaluation of the mobile nurse training (MNT) intervention – a step towards improvement in intrapartum practices in Bihar, India

**DOI:** 10.1186/s12884-017-1452-z

**Published:** 2017-08-23

**Authors:** Aritra Das, Dipty Nawal, Manoj Kumar Singh, Morchan Karthick, Parika Pahwa, Malay Bharat Shah, Tanmay Mahapatra, Kunal Ranjan, Indrajit Chaudhuri

**Affiliations:** 1 CARE India Solutions for Sustainable Development. H No. 14, Patliputra Colony, Patna, Bihar 800013 India; 2 Department of Epidemiology, Fielding School of Public Health, University of California – Los Angeles, 650 Charles Young Drive, South. Box 951772, Los Angeles, CA 90095 USA

**Keywords:** Obstetric care, Rural health, Nursing

## Abstract

**Background:**

Evidence shows that improving the quality of intrapartum care is critical for maternal survival. However, a significant rise in the proportion of facility-based births over the last decade in India - attributable to a cash transfer program - has not resulted in a corresponding reduction in maternal mortality, thanks, in part, to low-skilled care at facilities. The current study evaluated a mobile knowledge-based intervention aimed at improving quality of care by mentoring in-service staff nurses at public obstetric facilities.

**Methods:**

An independent evaluation team conducted baseline and post-intervention assessments at every facility using a mix of methods that included training assessments and *Direct Observation of Deliveries*. The assessment involved passive observation of pregnant women from the time of their admission at the facility and recording the obstetric events and delivery-related practices on a pre-formatted checklist-based tool. Maternal practices were classified into positive and negative ones and scored. Linear regression analysis was used to evaluate the association of MNT intervention with summary scores for positive, negative and overall practice scores. We evaluated retention of intervention effect by comparing the summary scores at baseline, immediately following intervention and 1 year after intervention.

**Results:**

In both unadjusted and adjusted analyses, the intervention was found to be significantly associated with improvement in positive practice score (Unadjusted: parameter estimate (β) = 16.90; 95% confidence interval (CI) = 15.20, 18.60. Adjusted: β = 13.14; 95% CI = 10.97, 15.32). The intervention was also significantly associated with changes in negative practice score, which was reverse coded to represent positive change (Unadjusted: β = 11.66; 95% CI = 10.06, 13.27. Adjusted: β = 2.99; 95% CI = 1.35, 4.63), and overall practice score (Unadjusted: β = 15.74; 95% CI = 14.39, 17.08; Adjusted: β = 10.89; 95% CI = 9.18, 12.60). One year after the intervention, negative practices continued to improve, albeit at a slower rate; positive labor practices and overall labor practice remained higher than the baseline but with some decline over time.

**Conclusions:**

Findings suggest that in low resource settings, interventions to strengthen quality of human resources and care through mentoring works to improve intrapartum maternal care.

**Electronic supplementary material:**

The online version of this article (doi:10.1186/s12884-017-1452-z) contains supplementary material, which is available to authorized users.

## Background

Globally, maternal mortality ratio (MMR) has been reduced by 43% since 1990 [1, 2]. However, this reduction, albeit impressive, falls short of the millennium development goal (MDG) regarding maternal health [2]. In 2015, as per the World Health Organization’s estimate, more than 300,000 maternal deaths took place owing to causes related to pregnancy and childbirth [1, 2]. It has been reported that intrapartum complications account for almost half of all maternal deaths [3]. Underlying conditions that may give rise to labor complications are often not anticipated beforehand and only become apparent at the time of delivery [4]. Therefore, for institutional deliveries, efficiency of facility-based management frequently play a crucial role in preventing maternal (and newborn) mortality and other adverse outcomes [4, 5].

Currently available evidence indicate that a major portion of childbirth-related maternal deaths are eminently avoidable through implementation of commonplace and affordable interventions [6]. Changing the place of delivery from home to a treatment facility and ensuring presence of a skilled person at the time of delivery have been suggested as the foremost interventions for improving pregnancy outcomes and reducing maternal mortality [4, 7, 8]. Low proportion of institutional deliveries under supervision of skilled birth attendants remains the principal reason behind the wide gap in maternal mortality ratio (MMR) between developed and developing worlds [1, 2, 9]. Unfortunately, until recent times, institutional deliveries constituted only a small proportion of all childbirths in India – only about 29% deliveries in rural India were carried out in a health facility during 2005–06 [10]. In order to address this gap, the Government of India launched an initiative called *Janani Suraksha Yojana* (JSY) in 2005 with the objective of promoting institutional deliveries and, in turn, reducing maternal and perinatal mortalities [8, 11]. This conditional cash transfer scheme, which incentivized mothers giving birth at a health facility, has led to a significant increase in the number of institutional deliveries during the recent years [8].

However, despite the rise in the proportion of institutional deliveries since the introduction of JSY, a commensurate decrease in MMR has not been observed [12, 13]. In a recent study, which utilized data from nine Indian states, Randive et al. [14] did not find any association between increase in institutional delivery proportions and MMR. This phenomenon is not unique to Indian context as prior reports revealed only a weak correlation between institutional deliveries and maternal mortality in developing nations, especially in countries with high MMR [15, 16]. It has been suggested that unless skills of the birth attendants and the quality of care at health facilities are improved, shifting to institutional deliveries, on its own, may not be able to bring significant changes in pregnancy outcomes [17–19]. Prior studies have reported that many harmful and unnecessary labor practices – such as drug-induced rapid augmentation of labor, application of manual fundal pressure, routine episiotomy etc. – have been prevalent in Indian obstetric care facilities [20–22]. Moreover, some essential components of intrapartum care e.g. monitoring of maternal blood pressure, cervical dilatation, and fetal heart rate are seldom practiced and postpartum women are often discharged prematurely [20, 21, 23]. In order to realize the full potential of institutional delivery promotion scheme the above issues need to be addressed.

The majority of deliveries occurring under JSY scheme in rural India are attended by nurses [17]. Therefore, systematic training aimed at enhancing the competence of nurses is an essential step towards improvement of quality of obstetric care at rural health facilities. In the present study, we sought to evaluate the impact of a ‘Mobile nurse training’ program on delivery-related practices in 80 facilities across eight districts of Bihar.

## Methods

### Design

The present study was implemented using a before-after design with the objective of evaluating the effectiveness of a nurse-mentoring intervention at the district and sub-district level state-run health facilities in Bihar, India. The before-after design was preferred to minimize the effect of any between-facility variations. Moreover, the mandate of the concerned nurse-mentoring intervention was to reach the facilities within a stipulated time. Therefore, a cluster randomized design was deemed infeasible. The main outcome of the study was the change in a set of evidence-based maternal care practices between the pre- and post-intervention periods.

### Intervention

In 2011, CARE India, in association with the State Government of Bihar (GoB) and under financial patronage of Bill and Melinda Gates Foundation, initiated the *Integrated Family Health Initiative (IFHI)*, a multifaceted program targeted at reducing mortality and malnutrition among infants and women of reproductive age in Bihar, a poor performing Indian state in terms of health and socio-developmental indicators [24]. *Mobile Nurse Training (MNT)* intervention [25] was a component of the IFHI that aimed to identify and address the gaps in the quality of intrapartum care at the facilities. The University of California San Francisco, PRONTO International and the University of Utah partnered with CARE India and the GoB to integrate their in-situ simulation and team training at important junctures of this intervention. The pilot phase of the MNT intervention was carried out in eight districts that were randomly selected from the 38 districts in Bihar. A preliminary assessment of all the government-run health centers/hospitals with functioning obstetric care facilities in these districts was carried out. Based on this initial assessment, 80 health centers/hospitals were purposively selected for the pilot phase.

Eight MNT teams – one for each IFHI district - each comprising of two highly-skilled nurses was constituted for every IFHI district. The nurse-trainers, selected from multiple Indian states through a rigorous interview process, were made acquainted with the training modules by a ‘Master trainer’ (M. Sc. in nursing). There was one Master Trainer for every two study district. As part of the intervention, each MNT team conducted one weeklong training workshop (or one round of training) at a selected facility and then moved on to another facility in the same district during the subsequent week. After 4 weeks, the MNT team revisited the first facility and the training cycle was renewed. Approximately six rounds of training were conducted at each facility and the training schedule continued in phases until all the facilities were covered. The initial rounds of training were mostly limited to performing various obstetric procedures on mannequins at the ‘Mini skills lab’ set up for the project. This was followed by hands-on training sessions, during which the MNT teams supervised the quality of care provided by the facility nurses at different stages – from admission of pregnant women to puerperium. Owing to lack of human resources, the MNT intervention could not be conducted at all 80 facilities simultaneously. During the initial round (between August, 2012 and June, 2013), 32 facilities received MNT, whereas 48 facilities were covered during the second round (from October, 2013 to April, 2014). Four additional MNT teams were employed (i.e. 12 teams in total) during the second round of interventions.

### Assessment

The *Direct Observation of Delivery (DOD)* study was undertaken to evaluate the effectiveness of the MNT intervention. In the DOD study, an independent evaluation team (DOD team), comprising of trained female study staff, performed the baseline (pre-MNT) assessment and the post-intervention (post-MNT) assessments at the study facilities. The DOD team members employed ‘direct observation’ methodology, whereby they passively observed the delivery-related practices at each facility. The duration of the assessment at each facility was 1 week, during which the DOD team members were required to observe minimum six normal vaginal deliveries (NVD). Only the NVDs conducted by the nurses were considered for assessment i.e. the information on deliveries conducted by doctors were not recorded. The period of assessment was increased if less than six eligible NVDs took place at a particular facility during the assessment week. During their period of assignment at a facility, DOD team members followed up every eligible pregnant woman from her time of admission at the facility till the time of her discharge/referral/death. The obstetric events and the delivery-related practices were recorded using a pre-formatted checklist-based tool. The tool (Additional file 1) was designed for the current study and included components from - 1) the Labour and Delivery Observation Checklist (JHPIEGO/Maternal Child Health Integrated (MCHIP) Program) and 2) WHO’s Safe Childbirth Checklist. In order to assess the applicability of the tool and detect problems with understanding of the items in the tool, it was field tested at two facilities in Saharsa, Bihar.

Baseline assessment in all 80 study facilities was carried out from August to November, 2012. Post-intervention assessment, for evaluation of the effectiveness of the MNT intervention, at any particular facility was conducted approximately 1 month after completion of intervention at that facility. Approximately 1 year after the conclusion of the training, a follow-up assessment was conducted in each of the 32 facilities that received the MNT intervention during the first round (between August, 2012 and June, 2013) to evaluate the retention of the intervention effect.

### Outcome measures – Labor practice indicators

The present analysis included 27 labor practice indicators – 21 of which were recommended (positive) practices, while rest six were unnecessary or harmful (negative) practices. The practices were selected based on literature review and opinion of an expert group. The DOD team members recorded whether a particular practice was performed during a delivery or not. The positive and negative practices are listed in Tables 2 and 3, respectively.

### Covariate measures

As the deliveries observed in the current study were not randomized, we included some delivery-related parameters in our analyses to control for potential confounding. These covariate measures were - type of facility, parity of the mother, time of delivery, and stage of labor at arrival at the facility. The selected facilities were classified into four broad categories – district hospital (DH), rural hospital (RH), sub-divisional hospital (SDH) and primary health centers (PHC). According to the hour of the day during which it took place, each delivery was categorized into ‘AM’ or ‘PM’ delivery.

### Statistical analyses

Descriptive analyses were carried out to determine the distribution of several facility- and program-related characteristics of the deliveries included in the DOD study. We performed before-after comparison of positive and negative labor practice indicators using the Cochran-Mantel-Haenszel chi-square test. We dichotomized the labor practices by giving each of them a score of 0 or 1. Presence of each positive practice and absence of each negative practice received a score of 1, while the reverse (absence of positive practice and presence of negative practice) got a score of 0. We created three summary scores for each observed delivery by adding the individual practice scores – i) positive practices summary score (21 items), ii) negative practices summary score (6 items), and iii) overall practices summary score (27 items). For making all the summary scores comparable and to aid interpretation, all summary scores were linearly transformed to a scale of 0–100. Simple and multiple linear regression analyses were performed with these summary practice scores to evaluate their associations with the MNT intervention (baseline = 0, post-intervention = 1). The summary practice scores were also utilized for evaluation of practice retention in the 32 facilities that received the MNT intervention during the first round. For these 32 facilities, we graphically compared the summary practice scores at three different time-points – at baseline, immediately following intervention and 1 year after intervention. We also performed Cochran-Armitage trend tests to evaluate presence of any trends in the summary practice scores, across the three time-points. All statistical analyses were performed using SAS 9.4 software package.

## Results

For estimation of the intervention effect, we used data from 1467 deliveries conducted under ‘direct observation’ in 80 obstetric care facilities – 842 deliveries from the period of baseline assessment and 625 deliveries conducted during post-intervention period. To assess retention of practices in 32 health facilities, we analyzed information on 710 deliveries – 219 at baseline, 249 immediately following intervention and 242 after one-year post-intervention. Regarding facility type, majority of the deliveries were carried out in PHCs (72%). Approximately one-third of the pregnant women (34%) received three antenatal check-ups, whereas 64% of the women received iron and folic acid supplementation during the antenatal period. Various individual- and program-related characteristics of the study participants are depicted in Table 1.

**Table 1:** Characteristics of the observed deliveries

Characteristic	Frequency	Percent (%)
No. of deliveries assessed during each assessment phase		
*Baseline assessment (80 facilities)*	842	49.27
*Post-intervention assessment (32 facilities)*	249	14.57
*Post-intervention assessment (48 facilities)*	376	22.00
*Follow-up assessment after 1 year (32 facilities)*	242	14.16
Type of facility/hospital		
*District hospital (DH)*	144	8.43
*Rural hospital (RH)*	168	9.83
*Sub-divisional hospital (SDH)*	166	9.71
*Primary health center (PHC)*	1231	72.03
District of residence of participating women		
*Patna*	235	13.75
*Samastipur*	196	11.47
*Begusarai*	216	12.64
*East Champaran*	254	14.86
*West Champaran*	208	12.17
*Saharsa*	187	10.94
*Khagaria*	160	9.36
*Gopalganj*	217	12.70
*Sheohar*	36	2.11
Mother originally planned to deliver the baby in		
*Hospital*	1623	94.97
*Home*	8	0.47
*No specific plan*	73	4.27
Mothers who received pregnancy related counselling from health workers^a^		
*ASHA*	1474	86.25
*AWW*	15	0.88
*ANM*	21	1.23
*Did not receive counselling*	191	11.18
Parity of participating mothers		
*0*	353	20.66
*1*	300	17.55
*2*	250	14.63
*3*	170	9.95
*4*	75	4.39
*5*	17	0.99
*> 5*	22	1.29
Did the mother receive iron and folic acid tablets during the current pregnancy		
*Yes*	1090	63.78
*No*	573	33.53
Did the mother receive at least one tetanus toxoid injection during the current pregnancy		
*Yes*	1666	97.48
*No*	31	1.81
No. of antenatal check-up received by mother		
*1*	1059	61.97
*2*	896	52.43
*3 or more*	579	33.88
Was the mother’s weight measured during antenatal check-up		
*Yes*	783	45.82
*No*	665	38.91
Was the mother’s height measured during antenatal check-up		
*Yes*	60	3.51
*No*	1354	79.23
Was the mother’s hemoglobin level assessed during antenatal check-up		
*Yes*	519	30.37
*No*	912	53.36
Did the mother undergo any urine test during antenatal check-up		
*Yes*	627	36.69
*No*	795	46.52
Was any ultrasonogram performed on the mother during antenatal check-up		
*Yes*	419	24.52
*No*	999	58.46
Did any ASHA/AWW/ANM^a^ workers visit the mother’s home during the current pregnancy		
*Yes*	1461	85.49
*No*	230	13.46

Observations with missing values excluded as applicable

The percentages may not add to 100% due to exclusion of missing values and rounding of numbers

^a^The following health workers provide pregnancy related counselling - Anganwadi workers (AWW), Accredited Social Health Activist (ASHA) and Auxiliary Nurse Midwives (ANM)

*Direct observation of Delivery* Study

Bihar, India, 2012–2014 [*N* = 1709]

Among the 21 positive labor practice indicators, 17 showed significant improvement in post-intervention assessment compared to baseline (Table 2). Among these, some crucial practices showed marked improvement in uptake proportions e.g. handwashing (69% vs 25%), wearing of gloves (91% vs 39%), administration of oxytocics for active management of third stage of labor (AMTSL) (66% vs 22%), controlled counter cord traction (71% vs 17%) etc. The intervention did not bring any significant differences in the uptake of fetal heart sound monitoring, cervical dilatation monitoring and use of sterile blade/scissors for cord-cutting. A statistically significant decrease was noted in performing test for urinary protein (2% vs 5%). Similar to the improvement noted for positive practices, four out of six negative labor practices decreased significantly following the MNT (Table 3). The practices that demonstrated reduction were – pubic hair shaving (6% to 2%), lithotomy position (32% to 15%), application of fundal pressure (40% to 10%), and vaginal packing (46% to 38%). There were no significant changes in the proportion of pregnant women who received routine enema and who had any substance applied to their cord stump.

**Table 2:** Comparison of ‘positive’ labor practices, before and after intervention, across all facilities

Practices	Pre-intervention (%) [*n* = 842]	Post-intervention (%) [*n* = 625]	*P*-value^a^
Blood pressure monitoring	126 (14.96)	165 (26.40)	**<0.0001**
Fetal heart sound monitoring	102 (12.11)	85 (13.60)	0.3989
Monitoring of cervical dilatation	739 (87.77)	539 (86.24)	0.3881
Blood test for hemoglobin	2 (0.24)	26 (4.16)	**<0.0001**
Urine test for protein	42 (4.99)	12 (1.92)	0.002
Handwashing by delivery attendant(s)	211 (25.06)	430 (68.80)	**<0.0001**
Wearing of gloves by delivery attendant(s)	331 (39.31)	567 (90.72)	**<0.0001**
Disposable delivery kit (DDK) or sterile tray used	141 (16.75)	237 (37.92)	**<0.0001**
Administration of oxytocics for AMTSL	185 (21.97)	410 (65.60)	**<0.0001**
Cord checked for pulsation before clamping	367 (43.59)	314 (50.24)	**0.0115**
Cord clamping after 2 min wait	582 (69.12)	530 (84.80)	**<0.0001**
Sterile cord clamp used	145 (17.22)	275 (44.00)	**<0.0001**
Sterile blade/scissors used for cutting the cord	568 (67.46)	433 (69.28)	0.4588
Controlled counter cord traction	143 (16.98)	446 (71.36)	**<0.0001**
Placenta checked for completeness	356 (42.28)	340 (54.40)	**<0.0001**
Membrane checked for completeness	325 (38.60)	281 (44.96)	**0.0144**
Genital tract exploration performed after delivery	685 (81.35)	563 (90.08)	**<0.0001**
Checked for perineal tear	563 (66.86)	483 (77.28)	**<0.0001**
Cleaning of the mother following delivery	791 (93.94)	604 (96.64)	**0.0181**
Use of standard recording format for labor events and parameters	197 (23.40)	355 (56.80)	**<0.0001**
Presence of qualified healthcare provider (doctor, trained nurse) at the time of delivery	791 (93.94)	610 (97.60)	**0.0008**

^a^Cochran-Mantel-Haenszel *X* ^*2*^

*AMTSL* Active management of third stage of labor

*P*-values in bold indicate statistically significant (*P* < 0.05) improvement following intervention

Bihar, India, 2012–2014 [*N* = 1467]

*Direct observation of Delivery* Study

**Table 3:** Comparison of ‘negative’ labor practices, before and after intervention, across all facilities

Practices	Pre-intervention (%) [*n* = 842]	Post-intervention (%) [*n* = 625]	P-value^a^
Pubic hair shaving of the mother	47 (5.58)	14 (2.24)	**0.0015**
Routine enema given	36 (4.28)	15 (2.40)	0.0526
Lithotomy position of the mother	273 (32.42)	92 (14.72)	**<0.0001**
Application of fundal pressure	419 (39.76)	61 (9.76)	**<0.0001**
Anything applied to cord stump	13 (1.54)	19 (3.04)	0.0525
Vaginal packing given	390 (46.32)	236 (37.76)	**0.0011**

^a^Cochran-Mantel-Haenszel *X* ^2^

*P*-values in bold indicate statistically significant (*P* < 0.05) decline following intervention

Bihar, India, 2012–2014 [*N* = 1467]

*Direct observation of Delivery* Study

Table 4 depicts the results of simple and multiple linear regression analyses to evaluate the association of the MNT intervention with the summary scores for labor practices. In both the unadjusted and adjusted analyses, the intervention was found to be significantly associated with improvement in the positive practice score (Unadjusted: parameter estimate (β) = 16.90; 95% confidence interval (CI) = 15.20, 18.60. Adjusted: β = 13.14; 95% CI = 10.97, 15.32). The intervention was also significantly associated with changes in the negative practice score, which was reverse coded to represent positive change (Unadjusted: β = 11.66; 95% CI = 10.06, 13.27. Adjusted: β = 2.99; 95% CI = 1.35, 4.63), and the overall practice score (Unadjusted: β = 15.74; 95% CI = 14.39, 17.08; Adjusted: β = 10.89; 95% CI = 9.18, 12.60).

**Table 4:** Parameter estimates from unadjusted and adjusted linear regression analyses to evaluate the association of ‘Mobile Nurse Training’ intervention with ‘Labor practice scores’. *Direct observation of Delivery* Study. Bihar, India, 2012–2014 [*N* = 1709]^a^

Scores	No. of items	Mean score (Standard deviation)		Unadjusted analysis		Adjusted analysis^c^	
		Pre-intervention [*n* = 842]	Post-intervention [*n* = 625]	Parameter estimate (β)	95% CI	Parameter estimate (β)	95% CI
Positive labor practices summary score^1^	21	41.81 (15.76)	58.70 (17.18)	16.90^b^	15.20, 18.60	13.14^b^	10.97, 15.32
Negative labor practices summary score^1^	6	76.68 (17.41)	88.35 (12.44)	11.66^b^	10.06, 13.27	2.99^b^	1.35, 4.63
Overall labor practices summary score^1^	27	49.56 (12.83)	65.29 (13.26)	15.74^b^	14.39, 17.08	10.89^b^	9.18, 12.60

^a^Observations with missing values excluded as and where applicable

Each practice amounted to a score of 1 (if present) or 0 (if absent). The negative practices were scored in reverse order (0 = present, 1 = absent). All summary scores were linearly transformed to a scale of 0–100

Positive parameter estimates indicate that the intervention is associated with improvement in labor practices and vice versa

^b^Statistically significant (*P* ≤ 0.05)

^c^Adjusted for type of facility, parity of the mother, time of delivery, and stage of labor at arrival at the facility

**Fig. 1:**
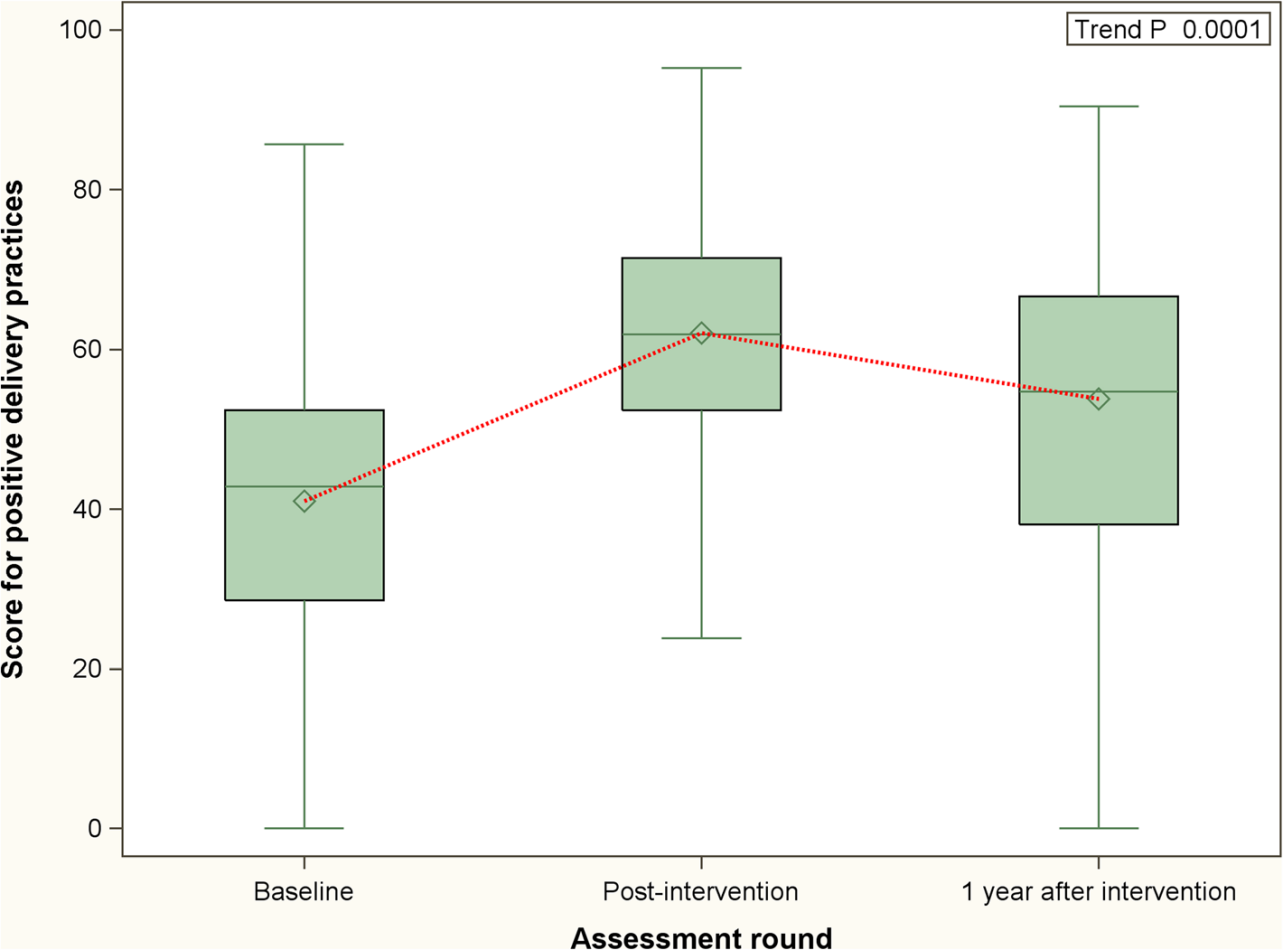
Distribution and periodic variation in positive delivery practices score (32 facilities) - before, immediately after and 1 year after intervention [Higher scores indicate increase in uptake of beneficial practices]

**Fig. 2:**
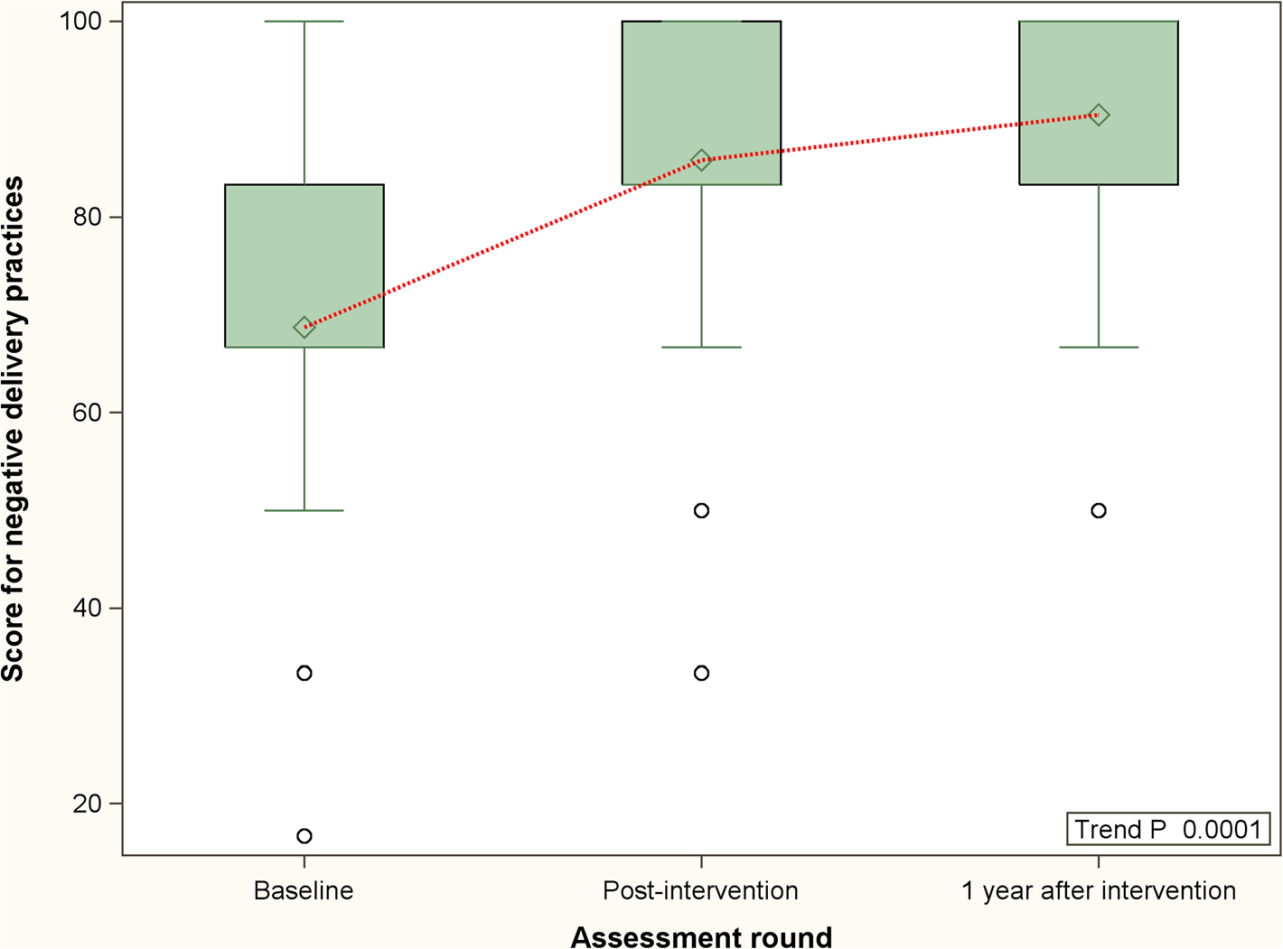
Distribution and periodic variation in negative delivery practices score (32 facilities) - before, immediately after and 1 year after intervention [Higher scores indicate increase in non-observance of harmful/unnecessary practices]

**Fig. 3:**
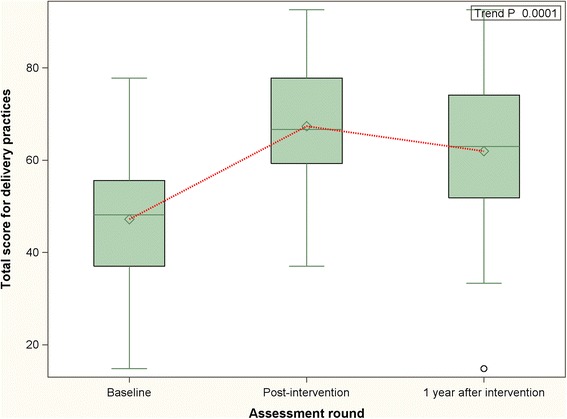
Distribution and periodic variation in overall delivery practices score (32 facilities) - before, immediately after and 1 year after intervention [Higher scores indicate improvement in practices]

Comparison of the summary scores for labor practices at different time points – baseline, immediate post-intervention and 1 year following intervention - are presented in the Figs. 1, 2 and 3. The summary scores in these figures pertain only to the 32 facilities that were covered during first round of the MNT. Compared to the period immediately following the intervention, a decrease was noted in the positive labor practice score 1 year after the conclusion of the MNT. However, despite the decrease, the positive practice score at 1 year post-intervention remained higher than that at baseline. This was also demonstrated by a statistically significant positive trend (trend *P* < 0.0001). Regarding the negative practice score, the score continued to improve even after cessation of the intervention, albeit at a slower rate (trend *P* < 0.0001). The temporal trend in the overall practice score was similar to the positive practice score – a decline was noticed at 1 year post-intervention but it remained higher than baseline (trend *P* < 0.0001).

## Discussion

In this pilot study conducted in Bihar, a resource-poor Indian state, we found that a composite training package aimed at improving the childbirth-related skill set of the facility nurses led to significant improvements in the uptake of essential delivery-related practices and reduction in unnecessary/harmful practices. The obstetric practices considered in our evaluation were key determinants of the quality of intrapartum care, and, in turn, maternal and perinatal outcomes. The encouraging findings of this study are indicative of a possible pathway to improve obstetric care services across India, especially in the rural and developmentally restricted regions.

We found that among the 21 recommended labor-related practices, 17 were delivered with a significantly greater frequency. Practices that did not show any significant change in uptake were fetal heart sound monitoring, cervical dilatation monitoring and use of sterile blade/scissors for cord-cutting. Based on our interactions with the nurses participating in the intervention, we found that many of them did not have any prior training or experience of monitoring fetal heart sounds. Although, training on fetal heart sound monitoring was part of the MNT, it seems that the short training was not enough to impart this demanding clinical skill to the nurses without any prior exposure. Monitoring of cervical dilatation was already a popular practice – monitored in about 88% deliveries observed in baseline assessment - and there was not much change following the intervention. Lack of change in the usage proportion of sterile blade/scissors, we believe, could be attributed to logistical issues rather than training performance. The only positive labor practice that showed significant reduction in post-intervention period was testing for urinary protein. Again, this finding could be attributed to logistical challenges, as testing kits were available only in a handful of facilities.

Regarding the harmful or unnecessary practices, four out of six negative labor practices were significantly reduced following the intervention. An important negative practice that demonstrated reduction was application of fundal pressure. Prior studies have reported labor augmentation methods e.g. fundal pressure and injection of oxytocics to be quite prevalent in health facilities across India [21, 23]. The birth attendants, reportedly, prefer deliveries to progress quickly and, consequently, change of labor augmentation practices often prove difficult [21]. In this context, reduction in the practice of fundal pressure application was an important success of the MNT intervention. Two negative practices for which no significant change was observed were routine enema and application of substances on cord stump. Both these practices had very low prevalence during the baseline assessment, and the intervention did not bring much change in these practices. The fact that these negative practices continue to persist is possibly indicative of the poor adoption of evidence-based nursing/midwifery practices in the public health facilities in India [26–28]. Also, the initiatives to increase familiarity with the existing evidence-based guidelines on delivery practices and ensuring adherence to such recommendations have also been found wanting [26]. Integrating instruction on guidelines as part of regular trainings, enhanced supervision of deliveries performed by nurses and implementation of possible disincentives (or incentives for adherence to recommended practices) could help in curbing the negative delivery practices [26].

Although the increased adoption of positive practices and reduction in negative practices were satisfactory as a whole, we hoped to achieve a more comprehensive change regarding some parameters. Even though most practices demonstrated significant improvement from baseline, uptake of some practices fell far short of achieving universality. Besides availability of the equipment and other logistical considerations, our informal communications with the nurses participating in MNT revealed two potential barriers to wider uptake of practices. First, although the rationale behind the recommended practices were discussed during the training, some nurses were not entirely convinced about the effectiveness of changed practices. These nurses probably felt more confident about following the prevailing practices or the practices they learned in their training schools. Second, some of the recommended practices meant that a considerably longer time had to be invested per delivery, which was not acceptable for everyone. As the current study was not designed to assess the above issues, a qualitative study could be undertaken for better identification of the barriers of practice adoption among nurses [29]. Also, remedial measures such as inclusion of a behavioral modification training module in MNT and performance-based incentives for nurses might facilitate practice adoption.

Comparison of the summary scores for labor practices in the immediate post-intervention period and after 1 year of cessation of the intervention revealed that there was slight decline in the frequency with which positive labor practices (and all practices combined) were delivered after 1 year. Importantly, despite the deterioration, adoption of the beneficial practices at 1 year remained significantly higher than that at baseline. Moreover, regarding non-observance of the negative practices, no decline was noted even after a gap of 1 year. These findings are crucial from the policy-makers’ perspectives as they demonstrate appreciable retention of knowledge (and practice) following the MNT intervention. We admit that, as with any other knowledge-based interventions, effectiveness of the MNT depends on reinforcements in the form of repeat training sessions. Also, refresher trainings at regular intervals would be essential for sustainability of achieved improvements. In this context, retention of training effects for a considerable period denotes reduced economic burden for the implementing agencies and, hence, improve the feasibility of large scale implementations. Also, as suggested in the literature, sustainability of this intervention is likely to improve further following its assimilation in existing organizational environment [30, 31].

The present study suffered from some important limitations. The before-after design was a major methodological limitation of this study. A controlled trial (preferably cluster randomized) design would have provided a much more robust assessment of intervention effect as that would have been free from confounding by temporal trends, if any. However, given the context of the study, it was not feasible to implement such a design. The interpretation of our findings depend on the assumption that, barring MNT, no major changes took place in the intervention facilities in the period between the baseline and the post-intervention assessments. Moreover, our evaluation of the intervention effect was limited to assessment of changes in various intrapartum practices. In absence of follow-up of individual deliveries, we could not assess the effect of intervention on distal outcomes such as reduction in maternal mortality. Therefore, we relied on the priori knowledge that changes in obstetric practices lead to improvement in maternal outcomes [4, 32, 33]. Further, as the assessments were carried out through ‘direct observation of deliveries’, *Hawthorne effect,* which has been defined as a phenomenon where modification in the behaviors of a population occurs as a result of the awareness of being under observation [34], could be another potential methodological limitation of the study. Prior studies have reported that Hawthorne effect affected the performance of health care providers [35–37]. However, even if *Hawthorne effect* was present, we postulate that it would have influenced the practices at both the baseline and the post-intervention assessments. Therefore, we assume that *Hawthorne effect* did not have much bearing on intervention effect. Also, the deliveries conducted during different times of the day might have had different levels of adherence to recommended delivery practices because of varying availability of skilled staff. Because of lack of statistical power, it was not possible to adjust for the hour of delivery in the analysis. Although, the analysis was adjusted for a dichotomous variable for delivery time (AM/PM), it might have been insufficient to account for the confounding introduced by time of delivery (if any). Finally, as the facilities for the MNT interventions were not selected randomly, we cannot comment on the generalizability of the study findings. However, as the present study included all types of primary and secondary care facilities that offered obstetric care in Bihar, we consider the results to be relevant in the context of Bihar, if not the whole of India.

## Conclusion

The limitations notwithstanding, the current study describes the evaluation of a large scale knowledge-based intervention targeted at nurses, often the sole skilled individual attending to institutional deliveries in Bihar and many other parts of India [17]. The elements of this unique intervention were selected not only based on literature review and the expert opinion but also involved inputs from the state health administrators to make them culturally suitable and increase their applicability. The fact that the MNT intervention was found to bring significant positive changes in quality of care in a large number of obstetric facilities across the eight intervention districts makes it a suitable candidate for future scale-up. Its case is further strengthened by demonstration of acceptable level of sustainability of effect. A concerted effort - including infrastructure development, political willingness, and advocacy for change - is needed if India wants to meet the Sustainable Development Goals regarding maternal health. The findings of this study suggest that MNT could be an important component of any comprehensive action plan aimed at fulfilling this objective.

## Additional file

Additional file 1: Direct Observation of Delivery tool. (DOCX 86 kb)

## Abbreviations

DH: District hospital (DH)
DOD: Direct observation of deliveries
IFHI: Integrated Family Health Initiative
IIHMR: Indian Institute of Health Management Research
JSY: Janani Suraksha Yojana
MDG: Millennium Development Goals
MMR: Maternal mortality ratio
MNT: Mobile nurse training
NVD: Normal vaginal delivery
PHC: Primary health center

## References

[1] WHO: Trends in Maternal Mortality: 1990 to 2015. In.: WHO, UNICEF, UNFPA, World Bank Group and the United Nations Population Division; Geneva; 2015.

[2] Maternal Mortality Fact Sheet. [http://www.who.int/mediacentre/factsheets/fs348/en/]. Accessed 26 Nov 2015.

[3] McClure EM, Goldenberg RL, Bann CM (2007). Maternal mortality, stillbirth and measures of obstetric care in developing and developed countries. Int JGynaecol Obstet.

[4] Campbell OM, Graham WJ (2006). Lancet maternal survival series steering g: **strategies for reducing maternal mortality: getting on with what works**. Lancet.

[5] Pasha O, Goldenberg RL, McClure EM, Saleem S, Goudar SS, Althabe F, Patel A, Esamai F, Garces A, Chomba E (2010). Communities, birth attendants and health facilities: a continuum of emergency maternal and newborn care (the global Network's EmONC trial). BMC pregnancy and childbirth.

[6] Hogan MC, Foreman KJ, Naghavi M, Ahn SY, Wang M, Makela SM, Lopez AD, Lozano R, Murray CJ (2010). Maternal mortality for 181 countries, 1980-2008: a systematic analysis of progress towards millennium development goal 5. Lancet.

[7] Bullough C, Meda N, Makowiecka K, Ronsmans C, Achadi EL, Hussein J (2005). Current strategies for the reduction of maternal mortality. BJOG.

[8] Lim SS, Dandona L, Hoisington JA, James SL, Hogan MC, Gakidou E (2010). India's Janani Suraksha Yojana, a conditional cash transfer programme to increase births in health facilities: an impact evaluation. Lancet.

[9] AbouZahr C (2003). Global burden of maternal death and disability. Br Med Bull.

[10] National Family Health Survey (NFHS-3), 2005–06: India. In*.*, vol. 1: International Institute for Population Sciences; Mumbai; 2007.

[11] Lahariya C (2009). Cash incentives for institutional delivery: linking with antenatal and post natal care may ensure ‘continuum of care’ in India. Indian J Community Med.

[12] Special bulletin on maternal mortality in India 2007–09 (SRS). In*.*: Office of registrar General and Census Commissioner, Ministry of Home Affairs, Government of India; New Delhi; 2011.

[13] Shah P, Shah S, Kutty RV, Modi D (2014). Changing epidemiology of maternal mortality in rural India: time to reset strategies for MDG-5. Trop Med Int Health.

[14] Randive B, Diwan V, De Costa A (2013). India's conditional cash transfer Programme (the JSY) to promote institutional birth: is there an association between institutional birth proportion and maternal mortality?. PLoS One.

[15] Graham WJ, Bell JS, Bullough CH. Can skilled attendance at delivery reduce maternal mortality in developing countries, vol. 17: ITG Press; Antwerp; 2001.

[16] Paxton A, Maine D, Freedman L, Fry D, Lobis S (2005). The evidence for emergency obstetric care. Int JGynaecol Obstet.

[17] Chaturvedi S, Upadhyay S, De Costa A (2014). Competence of birth attendants at providing emergency obstetric care under India's JSY conditional cash transfer program for institutional delivery: an assessment using case vignettes in Madhya Pradesh province. BMC pregnancy and childbirth.

[18] van den Broek NR, Graham WJ (2009). Quality of care for maternal and newborn health: the neglected agenda. BJOG.

[19] Friberg IK, Kinney MV, Lawn JE, Kerber KJ, Odubanjo MO, Bergh AM, Walker N, Weissman E, Chopra M, Black RE (2010). Sub-Saharan Africa's mothers, newborns, and children: how many lives could be saved with targeted health interventions?. PLoS Med.

[20] Iyengar SD, Iyengar K, Suhalka V, Agarwal K (2009). Comparison of domiciliary and institutional delivery-care practices in rural Rajasthan, India. J Health Popul Nutr.

[21] Iyengar K, Jain M, Thomas S, Dashora K, Liu W, Saini P, Dattatreya R, Parker I, Iyengar S (2014). Adherence to evidence based care practices for childbirth before and after a quality improvement intervention in health facilities of Rajasthan, India. BMC pregnancy and childbirth.

[22] Brhlikova P, Jeffery P, Bhatia GP, Khurana S (2009). Intrapartum oxytocin (mis) use in South Asia. J Health Stud.

[23] Spector JM, Agrawal P, Kodkany B, Lipsitz S, Lashoher A, Dziekan G, Bahl R, Merialdi M, Mathai M, Lemer C (2012). Improving quality of care for maternal and newborn health: prospective pilot study of the WHO safe childbirth checklist program. PLoS One.

[24] Integrated Family Health Initiative. Catalysing change for healthy communities. India: CARE India.

[25] Das A, Nawal D, Singh MK, Karthick M, Pahwa P, Shah MB, Mahapatra T, Chaudhuri I (2016). Impact of a nursing skill-improvement intervention on newborn-specific delivery practices: an experience from Bihar.

[26] Mirzabagi E, Deepak NN, Koski A, Tripathi V (2013). Uterotonic use during childbirth in Uttar Pradesh: accounts from community members and health providers. Midwifery.

[27] Chaturvedi S, Upadhyay S, De Costa A, Raven J (2015). Implementation of the partograph in India's JSY cash transfer programme for facility births: a mixed methods study in Madhya Pradesh province. BMJ Open.

[28] Brhlikova P, Harper I, Jeffery R, Rawal N, Subedi M, Santhosh M (2011). Trust and the regulation of pharmaceuticals: South Asia in a globalised world. Glob Health.

[29] Belizan M, Meier A, Althabe F, Codazzi A, Colomar M, Buekens P, Belizan J, Walsh J, Campbell MK (2007). Facilitators and barriers to adoption of evidence-based perinatal care in Latin American hospitals: a qualitative study. Health Educ Res.

[30] Hovlid E, Bukve O, Haug K, Aslaksen AB, von Plessen C (2012). Sustainability of healthcare improvement: what can we learn from learning theory?. BMC Health Serv Res.

[31] Franco L, Silimperi D, van Zanten T, MacAulay C, Askov K, Bouchet B, Marquez L. Sustaining Quality of Healthcare: Institutionalization of Quality Assurance. In*.*: Center for Human Services, Quality Assurance Project; Bethesda; 2002.

[32] Karolinski A, Micone P, Mercer R, Gibbons L, Althabe F, Belizan JM, Messina A, Lapidus A, Correa A, Taddeo C (2009). Evidence-based maternal and perinatal healthcare practices in public hospitals in Argentina. Int JGynaecol Obstet.

[33] Laopaiboon M, Lumbiganon P, McDonald SJ, Henderson-Smart DJ, Green S, Crowther CA, Group S-OS (2008). Use of evidence-based practices in pregnancy and childbirth: South East Asia Optimising reproductive and child health in developing countries project. PLoS One.

[34] Mayo E, Roethlisberger F, Dickson W (1939). Management and the worker: Cambridge.

[35] Davis DA, Thomson MA, Oxman AD, Haynes RB (1995). Changing physician performance. A systematic review of the effect of continuing medical education strategies. JAMA : the journal of the American Medical Association.

[36] Greco PJ, Eisenberg JM (1993). Changing physicians’ practices. N Engl J Med.

[37] Lohr KN, Brook RH, Kaufman MA: Quality of care in the New Mexico Medicaid program (1971-1975): the effect of the New Mexico experimental medical care review organization on the use of antibiotics for common infectious diseases. Med Care 1980, 18(1 Suppl):i-vi, 1-129. 6986518

