# Supplementary material for: Evaluation of the mobile nurse training (MNT) intervention – a step towards improvement in intrapartum practices in Bihar, India
Source: BMC Pregnancy Childbirth. 2017 Aug 23;17:266. doi: 10.1186/s12884-017-1452-z (PMC5569501; doi:10.1186/s12884-017-1452-z)
Supplement: Additional file 1: — Direct Observation of Delivery tool. (DOCX 86 kb) [file 12884_2017_1452_MOESM1_ESM.docx]

**Direct Observation of Delivery**

**Informed consent (Please fill separately for all section)**

| District _______________________________________________ | Block |
| --- | --- |
| Type of Facility __ | Name of Facility |
| Observation Start Date    D D M M Y Y Y Y | Observation Start Time AM / PM  HH MM |
| Observation End Date    D D M M Y Y Y Y | Observation End Time AM / PM  HH MM |
| Name of Pregnant/Delivered Women _____________________  _________________________________________________________ | Phone No of the  respondent __________________________ |
| (Ask to Asha, relatives or from labour room register)  Address of the respondent_______________________________________________________________________ | |
| Name of the Asha | Phone no of Asha |

**Informed Consent:**

My name is…………………………………. I work with CARE India, an NGO, and I work with the Grade As and other (Specify) staff at this hospital. I support them in providing better care to patients.

Today, I will seek information from this and previous pregnancies and deliveries. We will use this information to further improve services at this and other (Specify) hospitals.

May I ask you questions and remain with you during your stay in the hospital?

Before we start, do you want to ask me any questions? Yes………………….1 No… 2

| **Case observed from:**  Arrival 1  Admission 2  In maternity ward, before delivery 3  Move to labour room 4  In labour room, before delivery 5 | Name of the ANM / GNM /LHV/ DR who has conducted delivery  Phase………………….  Type of assessment?  Pre Assessment… ..1  Post Assessment… .2 |
| --- | --- |
| **Case observed until:**  In labour room, after delivery 1  In maternity ward/ postnatal ward, after delivery 2  Refer 3 |  |

Name of the observer_________________________ Signature of the observer__________________

Name of the editor___________________________ Signature of the Editor_____________________

Date_______________________________________

| **Sr.No.** | **Question** | **Answer** | **Comment** | **Skip** |
| --- | --- | --- | --- | --- |
| **Fill out this section for any woman who is in labor who is likely to have a vaginal occipital delivery. Record observations until the time the woman is transferred from the labour room to the maternity or post-delivery ward, or until you need to leave the PHC, whichever is earlier. Ensure that the "Basic Information" section is also filled in.** | | | | |
| 101 | Time observation began: | HOURS  AM/PM  MINUTES |  |  |
| 102 | Time of arrival of mother in labor room was recorded | Yes 1  No 2 |  |  |
| 103 | Stage of labor on arrival at the facility | First Stage 1  Second Stage 2  Third Stage 3 |  |  |
| 104 | Was this a vaginal, occipital delivery? | Yes 1  No 2 | **(If N, make separate notes instead of using this tool)** | **If no stop here** |
| 105 | Mother well covered when she entered the labor room? | Yes 1  No 2 |  |  |
| 106 | Initial assessment in labor room done by | Doctor 1  Nurse 2  Mamta 3  ASHA 4  Other 5  Not done 6 |  |  |
| 107 | Were any of the following examined while the mother was in the labour room? | YES NO  Temperature 1 2  Pulse 1 2  Blood pressure 1 2  Uterine contraction 1 2  Fetal heart rate 1 2  Cervical dilatation 1 2  Station 1 2 |  | Write in Table no 1 |
|  |  |  |  | write in Table no 2 |
| 108 | Were standard recording formats used for recording the above? | Yes 1  No 2 |  |  |
| 109 | Were standard recording formats used for recording the above? | YES NO  A. Hemoglobin 1 2  B. Urine protein 1 2  C. Urine sugar 1 2  D. Blood sugar 1 2  E. Blood group 1 2  F. HIV 1 2  G. VDRL 1 2  H. Other (Specify) 1 2  I. Other (Specify) 1 2 | Investigations on arrival for Delivery |  |
| 110 | Did the mother change to a formal hospital patient dress? | Yes 1  No 2 |  |  |
| 111 | Perineal shaving done? | Yes 1  No 2 |  |  |
| 112 | Enema given? | Yes 1  No 2 |  |  |
| 113 | Was any vaginal examination performed while mother was in the labor room? | Yes 1  No 2 | If Y, enter details in the vaginal examination chart | Write in Table No 2 |
| 114 | Cauterization done? | Yes 1  No 2 |  |  |
| 115 | Any medication given before full dilatation? | Yes 1  No 2 | If Y, enter details in medication chart | Write in Table No 3 |
| 116 | Time when full dilatation / start of second stage of labour was recorded | Yes 1  No 2 |  |  |
| 116.A | If Yes, Time of full dilatation | HOURS  AM/PM  MINUTES |  |  |
| 117 | Number of qualified attendants (nurses + doctors) in attendance during second stage: | Nurse    Doctor |  | If no one is available go to 19 |
| 118 | Did at least one attendant do the following? "Yes" must be based on observation.  A. Put on plastic apron  B. Put on mask  C. Put on cap  D. Hand-wash | YES NO  A. Put on plastic apron 1 2  B. Put on mask 1 2  C. Put on cap 1 2  D. Hand-wash 1 2 |  |  |
|  | E. Wear gloves | In One Hand 1  In Tow Hands 2  Not Wear 3 | If Y, enter details in Handwashing section | Go to section 5 |
| 119 | Were the mother's legs well-supported? | Yes 1  No 2 |  |  |
| 120 | Were the mother's legs tied? | Yes 1  No 2 |  |  |
| 121 | Painting of part done with antiseptic solution? | Yes 1  No 2 |  |  |
| 122 | Mother draped? | Yes 1  No 2 |  |  |
| 123 | Did the attendant correctly explain to the mother when and how to push down? | Yes 1  No 2 |  |  |
| 124 | Was fundal pressure applied anytime during labor? | Yes 1  No 2 |  |  |
|  | A. By whom? | Doctor 1  Nurse 2  ASHA 3  Mamta. 4  Other 5 |  |  |
| 125 | New DDK used? | Yes 1  No 2 |  |  |
| 126 | Was a sterile delivery tray opened for use at the time of delivery? | Yes 1  No 2 |  |  |
| 127 | Cervical sweeping done? | Yes 1  No 2 |  |  |
| 128 | Episiotomy done? | Yes 1  No 2 |  |  |
|  | A. Local anesthesia given? | Yes 1  No 2 |  |  |
|  | B. Sterile episiotomy tray used? | Yes 1  No 2 |  |  |
| 129 | Perineal support given when head delivered? | Yes 1  No 2 |  |  |
| 130 | Checked for cord around the neck? | Yes 1  No 2 |  |  |
|  | A. If Yes, cord around the neck Present | Yes 1  No 2 |  |  |
|  | A.1. If Yes, cord clamped and cut immediately | Yes 1  No 2 |  |  |
| 131 | Did attendant record time of birth? | Yes 1  No 2 |  |  |
| 131.A | If yes, record Time | HOURS  AM/PM  MINUTES |  |  |
| 132 | Temperature in labour room at birth: | comfortable 1  cold 2  warm 3 |  |  |
| 133 | Birth status: | Live Birth 1  Still Birth 2 |  | If Dead (Still) birth baby, go to 39 |
| 134 | Baby placed on mother's abdomen immediately? | Yes 1  No 2 |  |  |
| 135 | Baby wiped dry? | Yes 1  No 2 |  |  |
| 136 | Baby wrapped in clean, dry cloth? | Yes 1  No 2 |  |  |
|  | A. Cloth brought by patient or hospital linen? | Patient 1  Hospital 2 |  |  |
| 137 | Baby cried immediately? | Yes 1  No 2 | **If N, use asphyxia section for recording observations related to resuscitation** |  |
| 138 | Cord checked for pulsations before clamping? | Yes 1  No 2  NA 3 |  |  |
| 139 | Sterile clamp used? | Yes 1  No 2 |  |  |
| 140 | New / sterile blade / scissors used to cut cord? | Yes 1  No 2 |  |  |
| 140.A | Record Time | HOURS  AM/PM  MINUTES |  |  |
| 141 | Disposable sterile plastic cord clamp used? | Yes 1  No 2 |  |  |
| 142 | Anything applied to cord stump? | Yes 1  No 2 |  | If No, go to 43 |
|  | A. If Y, what applied to cord stump? | mustard oil 1  Dung 2  Vermilion 3  talcum powder 4  Gentian violet paint 5  Other 6 |  |  |
| 143 | Eyes wiped with separate sterile gauze for each eye? | Yes 1  No 2 |  |  |
| 144 | Skin-to-Skin Care initiated? | Yes 1  No 2 |  |  |
| 145 | Baby's head covered? | Yes 1  No 2 |  |  |
| 146 | Was the mother provided guidance and support for Breastfeeding? | Yes 1  No 2 |  |  |
| 147 | Breastfeeding initiated? | Yes 1  No 2 |  |  |
| 148 | Oxytocic given during delivery? | Yes 1  No 2 | Inj Oxytocin / Tab Misoprostol / Ing Methergine / Tab Methergine (Details in drug table) | write in Table No 3 |
|  | A. When administered? | Before delivery of anterior shoulder 1  At delivery of anterior shoulder 2  Within a minute after delivery of baby but before delivery of placenta 3  More than one minute after the delivery of baby but before the delivery of placenta 4  After the delivery of placenta 5 |  |  |
| 148 | Uterine massage done? | Yes 1  No 2 |  |  |
| 150 | Cord traction used? | Yes 1  No 2 |  | If No go to 51 |
| 150.A | Record Time | HOURS  AM/PM  MINUTES |  |  |
| 150.b | Control Counter cord traction used? | Yes 1  No 2 |  |  |
| 151 | Placenta delivered time: | HOURS  AM/PM  MINUTES |  |  |
| 152 | Placenta checked for completeness? | Yes 1  No 2 |  |  |
| 153 | Membrane checked for completeness? | Yes 1  No 2 |  |  |
| 154 | Was genital tract exploration performed after delivery? | Yes 1  No 2 |  | If No, go to 55 |
|  | If yes, how? | ________________________________________  ________________________________________ |  |  |
|  | A. Vaginal exploration (by finger) | Yes 1  No 2 |  |  |
|  | B. Cervical exploration (by instruments) | Yes 1  No 2 |  |  |
|  | C. Exploring uterine cavity (by introducing a hand in the uterus) | Yes 1  No 2 |  |  |
| 155 | Vaginal packing done? | Yes 1  No 2 |  |  |
| 156 | Checked for perineal tear? | Yes 1  No 2 |  | If No, go to 57 |
| 156.A | Perineal tear present? | Yes 1  No 2 |  | If No, go to 57 |
| 156.B | Repaired perineal tear? | Yes 1  No 2 |  | If No, go to 57 |
| 156.C | Who repaired perineal tear? | Doctor 1  Nurse 2  Other 3 |  |  |
| 157 | Episiotomy sutured? | Yes 1  No 2  NA 3 |  | If No or NA, go to 59 |
|  | A. Sutured by whom? | Doctor 1  Nurse 2  Other 3 |  |  |
| 158 | Focused light was available at the time of perineal/ episiotomy repair? | Yes 1  No 2  NA 3 |  |  |
| 159 | Checked for amount of blood loss? | Yes 1  No 2 |  |  |
| 160 | Cleaning of mother done at the end of delivery? | Yes 1  No 2 |  | If No, go to 61 |
| 160.A | A. Who cleaned the mother? | Nurse 1  Mamta 2  Patient's relative 3  Other 4 |  |  |
| 161 | Sanitary pad provided to the mother? | Yes 1  No 2 |  |  |
| 162 | Was the mother in lithotomy position after the procedure was finished? | Yes 1  No 2 |  |  |
| 163 | Mother well covered after the delivery? | Yes 1  No 2 |  |  |
| 164 | Delivery summary noted in case paper | Yes 1  No 2 |  |  |
| 165 | Birth of baby registered in birth/ delivery register | Yes 1  No 2 |  |  |
| 166 | Privacy maintained during delivery process in labor room | Yes 1  No 2 |  |  |
| 167 | Birth companion present at the time of delivery? | Yes 1  No 2 |  |  |
| 168 | Mother well covered while moving from the labor room to maternity ward? | Yes 1  No 2 |  |  |
| 169 | How was mother transferred from labor room? | By walking 1  On stretcher 2  In wheelchair 3 |  |  |
| 170 | Baby was taken out of labor room along with the mother | Yes 1  No 2  NA 3 |  |  |
| 171 | Was cord cut again | Yes 1  No 2  NA 3 |  |  |
|  | A. If Yes, What used to cut cord? | ________________________________________  ________________________________________ |  |  |
|  | **Summary Questions :** | | |  |
| 1. | Blood pressure measured | Yes 1  No 2 |  |  |
| 2. | FHS monitored | Yes 1  No 2 |  |  |
| 3. | Cervical dilatation monitored | Yes 1  No 2 |  |  |
| 4. | Hemoglobin done | Yes 1  No 2 |  |  |
| 5. | Urine protein done | Yes 1  No 2 |  |  |
| 6. | Attendant washed hands | Yes 1  No 2 |  |  |
| 7. | Attendant wore gloves | Yes 1  No 2 |  |  |
| 8. | Fundal pressure applied | Yes 1  No 2 |  |  |
| 9. | DDK/ sterile delivery tray used | Yes 1  No 2 |  |  |
| 10. | Skin-to-skin care initiated immediately after birth | Yes 1  No 2 |  |  |
| 11. | Cord clamped 2 minutes or more after the delivery | Yes 1  No 2 |  |  |
| 12. | Anything applied to cord stump | Yes 1  No 2 |  |  |
| 13. | Oxytocic correctly administered for AMTSL | Yes 1  No 2 |  |  |
| 14. | Sanitary pad provided to mother | Yes 1  No 2 |  |  |
| 15. | Breastfeeding initiated in labor room | Yes 1  No 2 |  |  |
| 16. | Standard recording format used | Yes 1  No 2 |  |  |
| 17. | Qualified provider present at the time of delivery | Yes 1  No 2 |  |  |
